# Colour and chemical stability of bismuth oxide in dental materials with solutions used in routine clinical practice

**DOI:** 10.1371/journal.pone.0240634

**Published:** 2020-11-11

**Authors:** Josette Camilleri, Joseph Borg, Denis Damidot, Enrico Salvadori, Peter Pilecki, Paul Zaslansky, Brian W. Darvell

**Affiliations:** 1 School of Dentistry, University of Birmingham, Birmingham, United Kingdom; 2 Systems Engineer, Triq L-Uqija Ta L-Ibrag, Is-Swieqi, Malta; 3 Department of Civil and Structural Engineering, Faculty of Civil Engineering, Ecole de Mines, LGCgE-GCE, Douai, France; 4 Department of Chemistry, University of Torino, Torino, Italy; 5 Imaging and Microscopy, King’s College London, London, United Kingdom; 6 Department for Operative and Preventive Dentistry, Centrum für Zahn-, Mund- und Kieferheilkunde, Charité - Universitätsmedizin Berlin, Berlin, Germany; Danube Private University, AUSTRIA

## Abstract

Bismuth(III) oxide is included as a radio-opacifier in dental materials, including hydraulic silicate cements, the material of choice for several endodontic procedures. It has been implicated in tooth discoloration after contact with endodontic irrigants, in particular NaOCl solution, To date, there has been no work on the chemistry: all reports have been of clinical findings only. The purpose now was to report the reactions leading to colour change from Bi_2_O_3_ in contact with solutions used in routine endodontic practice. Ten-gram portions of Bi_2_O_3_ were immersed in either water, NaOH, NaCl, NaOCl or HCl solution, either in the dark or exposed to visible light, and samples retrieved at 1, 4, 12 and 24 weeks. After washing, these were exposed to either added CO_2_ or not, for 1 week while drying, and under the same dark or light conditions. Changes in appearance were monitored by photography and colour measurement, and chemically by X-ray diffraction and Fourier-transform infrared spectroscopy. 24-week material was studied using electron paramagnetic resonance and Raman spectroscopy; NaOCl-treated material was also examined by scanning electron microscopy. With water, NaCl and NaOH, bismuth subcarbonate was formed. With or without added carbon dioxide, discoloration occurred from pale yellow to light brown when exposed to light, and to a lesser extent in the dark, intensifying with time. In contrast, exposure to NaOCl rapidly formed a dark brown-black sodium bismuthate. With HCl, white BiOCl was formed. Bi_2_O_3_ is not at all inert in this context as is commonly believed, denying its principle of use. Previously unreported solution-mediated reaction occurs readily even in water and NaCl solution, forming new compounds that discolour. In contact with NaOCl sodium bismuthate is formed; severe darkening occurs rapidly. The reactivity is such that Bi_2_O_3_ is not indicated for dental materials and should be withdrawn from use.

## Introduction

Materials and devices that are implanted in the body, including for dentistry, are ordinarily required to be radio-opaque to allow radiographic detection (by contrast with adjacent tissues), thus permitting clinical follow-up. Indeed, radio-opacity is important for medico-legal reasons as any material implanted in the body needs to be detectable after placement. If the material is not naturally radio-opaque it may be made so by the addition of a radio-opacifier, typically as a dispersed powder. Agents commonly used include barium sulfate, calcium tungstate, and zirconium, tantalum and bismuth oxides. Radio-opacifiers are expected to be inert, neither reacting with any component of the host material nor interfering in any setting reaction, let alone reacting with the material’s environment, *i*.*e*., the patient’s tissues.

Bismuth(III) oxide (Bi_2_O_3_) is used as radio-opacifying agent in hydraulic silicate cements (HSCs) because of bismuth’s high atomic number (Z = 83) and the low atomic numbers of the elements present otherwise. Bi_2_O_3_ is commonly described as being insoluble in water, and unreactive at pH > 7 [1], while HSCs, having excess calcium hydroxide when set, have a pH ~ 12 [2]. HSCs are used for a number of root canal treatment procedures. These include pulp therapy and regenerative treatments where materials come into contact with the tooth crown. HSCs are also used as root canal sealers, perforation repair and root-end filling materials in surgical procedures and thus placed subgingivally.

**Fig 1:**
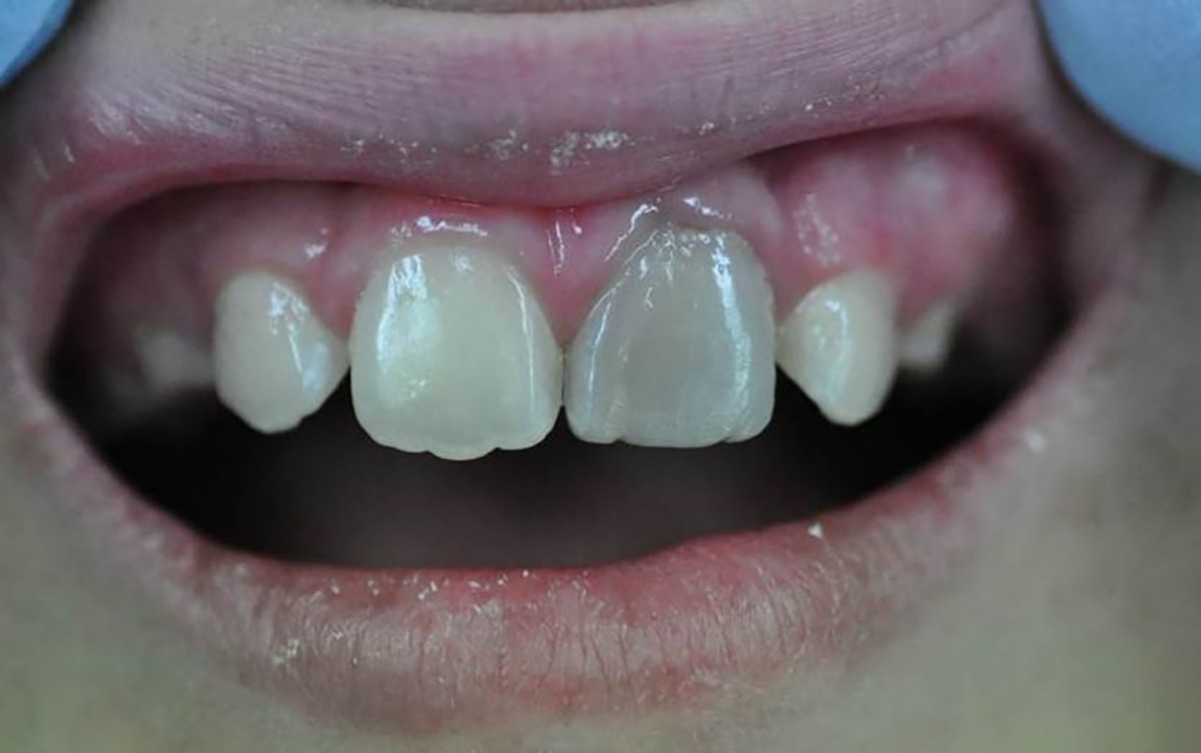
Tooth discolouration attributed to the use of “MTA”. Photo: Bill Kahler, University of Adelaide.

Bi_2_O_3_ has been implicated in tooth discoloration (Fig 1), associated with the use of both ‘Gray MTA’ and ‘White MTA’ (ProRoot MTA, Dentsply-Sirona, Tulsa, OK, USA), which are types of HSC; most reports have been of incidental findings [3–8]. For example, Gray MTA led to clinically-perceptible crown discoloration after 1 month, whilst that with White MTA was visually detectable after 3 months [9], suggesting that the use of these products in teeth that are ordinarily visible (*e*.*g*., on smiling) should be avoided. Causative factors suggested for the reported changes in colour of bismuth oxide from yellow to dark brown have included sodium hypochlorite [10], formaldehyde [11], contact with collagen, as in tooth tissue [12], and blood [13, 14]. A specific test of the effect of sodium hypochlorite residue in contact with tooth structure gave appreciable discolouration over that of the material alone [15]. Exposure to light and a lack of oxygen have also been claimed to be precipitating factors in such discoloration [16, 17]. All of these conditions are clinically relevant. Sodium hypochlorite is used as a dentine disinfectant in the management of dental caries, being suggested for this role in clinical guidelines published by the European Society of Endodontology [18] as it improves the adaptation of hydraulic cements to the substrate in caries-affected dentine [19]. It is effective in the dissolution of organic matter; deproteinization of dentine is recommended prior to cavity restoration with resin composites [20, 21]. In endodontic applications, it is also used as an irrigant in root canal therapy [22, 23].

Anterior teeth in particular are ordinarily exposed to light, but all teeth may be exposed to intense blue light when light-cured resins are used as tooth-coloured restorations. Carbonation of HSCs in contact with blood and tissue fluids has been demonstrated [13, 16, 17], making carbon dioxide exposure a further putative factor. However, no reactions have been suggested for the bismuth oxide discolouration and the chemistry of the process(es) involved remains obscure. Since enamel is somewhat translucent, any remnant of coloured material, any change in the colour of underlying material, or material diffusing to and deposited in it as a coloured substance, can be deleterious to appearance.

The dental procedures in which these materials are used, are routine and undergone by many people on a daily basis. If a tooth that has undergone root canal (endodontic) treatment becomes discoloured it may necessitate further management even in the absence of any sign of infection. This might involve bleaching, or partial (*i*.*e*. a veneer) or full coverage of the tooth (*i*.*e*. a crown), with the implied further intervention, extra expense for the patient and risk of retribution and litigation for the clinician. Importantly, discoloration due to heavy metals is not amenable to bleaching as only organic chromophores can be susceptible.

Given that most of the above reports of discoloration, whilst evidential, were essentially anecdotal and speculative as to cause, this study was undertaken to investigate whether indeed bismuth oxide is capable of reaction under clinically relevant conditions, and if so what chemistry might be involved, and whether such chemistry is associated with colour changes. Given the use of water, saline and hypochlorite as endodontic irrigants, a systematic series of reagents involving chloride, hypochlorite (most particularly) and sodium ions, and the factors of the presence or absence of light and carbon dioxide, were tested with a view to identifying means of avoidance or mitigation.

## Materials and methods

The materials and reagents used together with manufacturer details are given in Table 1. Bismuth(III) oxide (‘Reagent Plus’, 99.9%, 223891; Sigma Aldrich, Gillingham, Kent, UK) was characterized before and after contact with either water (as a control), sodium hydroxide, sodium chloride, sodium hypochlorite or hydrochloric acid solutions (Table 1) to test the effect of ions that might be encountered in practice in treatment. The NaCl (‘normal saline’) and NaOCl solutions were similar to endodontic irrigants, but sodium and chloride are also normal physiologically-relevant ions. It should be noted that NaOCl solution has chloride present because of the manner of manufacture, and it is also stabilized against the generation of chlorine by the addition of NaOH, to ensure pH > 11. The NaOH and NaCl solutions thus provide additional controls.

**Table 1:** Treatment media.

Medium	Concentration	Source	pH
**Deionized water**	-	Elgastat Optima, BioNordikaBergman, Oslo, Norway	6.8 ± 0.3
**Sodium hydroxide**	1 mol/L	71463, Fluka Chemie, Buchs, Switzerland	12.8 ± 0.1
**Sodium chloride**	0.9 mass %	3010337, Versol, Lyon, France	7.4 ± 0.2
**Sodium hypochlorite**	10%	71696, Sigma Aldrich, St Louis, MO, USA	12.4 ± 0.1
	(by redox titration)		
**Hydrochloric acid**	1 mol/L	H/1200/PB15, Fisher Scientific, Hampton, NH, USA	0.4 ± 0.1

The purity of the oxide was assessed by X-ray fluorescence (XRF) (S4 Pioneer; Bruker, Billerica, MA, USA). Approximately 4 g of the powder (< 75 μm) were mixed with lithium tetraborate (222534; Sigma-Aldrich) as flux in the ratio of 1:5 by mass, fused at 900 ~ 1000°C in a platinum crucible, and cast into a circular platinum alloy mould with a flat bottom.

The pH of each medium was as-supplied, and for clinical irrigants as in ordinary use. Values were determined in triplicate before use (at 20°C) with a glass probe (HI 1230), single-junction (Ag/AgCl) ceramic reference electrode (HI 1131) and pH meter (Hanna HI 3221; all Hanna Instruments, Woonsocket, RI, USA) which had been calibrated at three points (pH 4.00, 7.00 and 10.00) using standard calibrating solutions (Scharlau; Scharlab, Sentimenat, Spain) with temperature compensation (HI 7662; Hanna Instruments). Mean values (and standard deviations) are shown in Table 1.

### Test medium exposures

Ten-gram portions of bismuth oxide were each immersed in ~40 mL of the test media at 23°C in filled screw-capped 40-mL polypropylene containers (Labplex LXP40; Sterilin, Newport, Wales) agitated manually for 1 min for initial full dispersion then allowed to settle and stand undisturbed. No attempt was made to exclude CO_2_ from those media. Four groups were created each with 5 test solutions. Two groups were kept in an airtight transparent box (~20 × 15 × 15 cm^3^), exposed to daylight, with two low-heat halogen lamps (Decostar 51 PRO 24°, 50 W; Osram, Augsburg, Germany) positioned some 30 cm above. The containers of the other two groups were individually wrapped in aluminium foil, and kept in a black box, to exclude light. At each sampling time (1, 4, 12 and 24 weeks, based on the time-scale of other discoloration observations [10, 24], plus longer terms) approximately 1 g was retrieved from each solution, drained, washed with deionized water (DI) (Elgastat Optima; BioNordikaBergman, Oslo, Norway) by decantation three times, and placed in an open glass 10 mL vessel. These samples were then stored for 1 week, in the presence of silica gel as slow desiccant, under the following conditions:

1. In the dark, without CO_2_ (D−)
  In a vacuum desiccator; carbon dioxide was absorbed by the use of a 10 g portion of soda lime (8001308; Breckland Scientific, Stafford, UK) in a glass petri dish, and placed in a light-tight black box. This is the treatment control set.
2. In the dark, with added CO_2_ (D+)
  As for D−, but at atmospheric pressure, with CO_2_-enrichment (BD GasPak 260679; Becton Dickson, Sparks, MD, USA) (no soda-lime). The concentration was not measured.
3. Exposed to light, without CO_2_ (L−)
  As for D−, but with lighting as above.
4. Exposed to light, with added CO_2_ (L+)
  As for L−, but with CO_2_ added as for D+.

**Fig 2:**
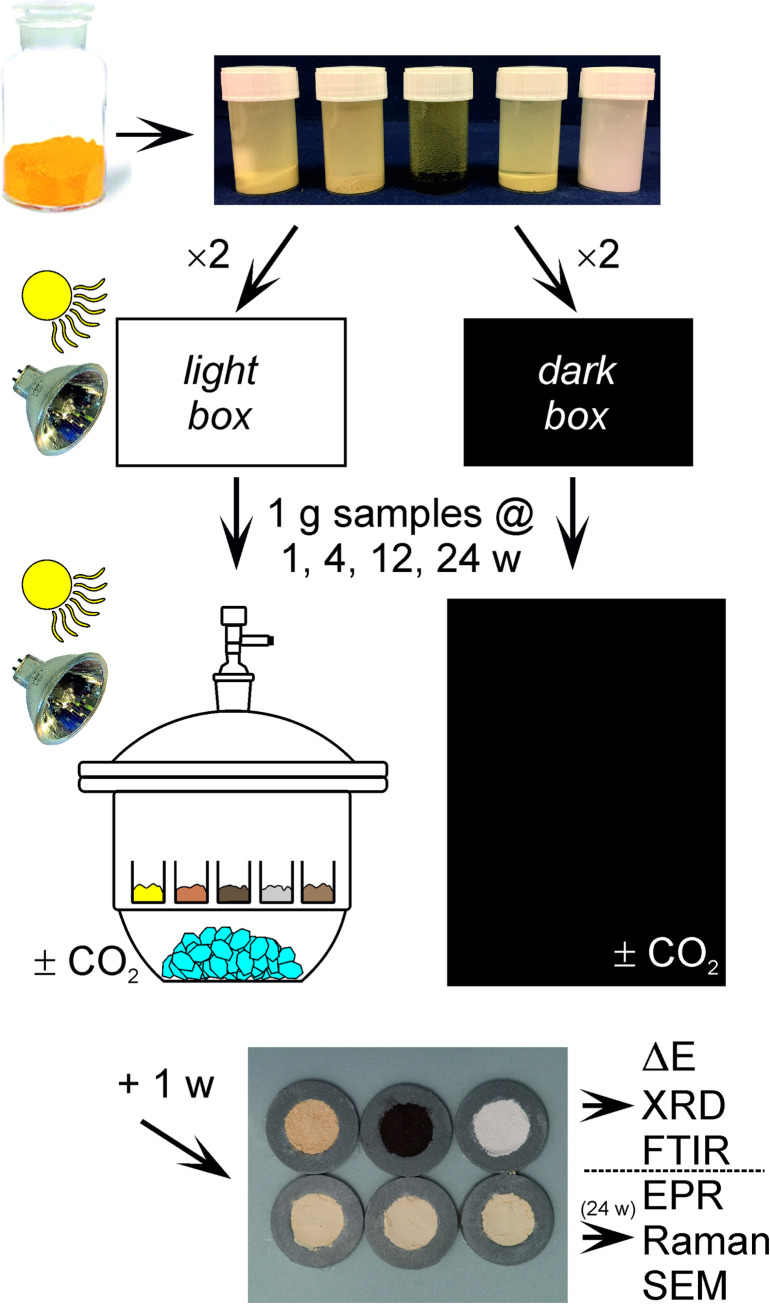
Experimental scheme outline.

At the end of that one week, all treated material was subject to standardized photography, colour measurement, X-ray diffraction (XRD), and Fourier-transform infrared spectroscopy (FTIR). The general scheme is outlined in Fig 2.

An additional 1 g portion of as-supplied material was exposed to light as above, and one kept dark, in laboratory air as additional controls for photography and colour measurement at 24 weeks (as detailed below).

### Sample photography

Samples of each of the eight treated powders were held, levelled, in 10-mm ring moulds, in a group on a light grey background, for each exposure period and photographed (D800e; Nikon, Tokyo, Japan), using a macro lens (Sigma 105 mm F/2.8 EX DG; Nikon), vertically using a tripod (Eddie; 3 Legged Thing, Stagsden, UK) inside a light diffuser (Lastolite Cubelite 90 cm; Manfrotto Lighting, Ashby-de-la-Zouch, UK). In addition to ambient natural indoor light, four halogen lamps (Decostar 51 PRO 24°, 50 W; Osram) were arranged to illuminate the outside of the diffuser. The remote shutter release was half-pressed to acquire focus, then fully depressed to acquire the image, with the camera in exposure-delay mode, to avoid vibration caused by mirror lock-up, using f/8, ISO 100, shutter speed 1/25, matrix metering, and an exposure bias of 1 EV in camera plus 0.3 EV in post-processing (on a computer), when the colour temperature was adjusted to lie between 3100 K and 3300 K. Images were saved in JPEG format.

### Spectrophotometry

Colour was assessed using a spectrophotometer (CM-50Bi; Minolta. Tokyo, Japan), using the L*a*b* coordinate system [25]. The colour difference (ΔE) with respect to the untreated control was calculated:

$$\Delta E={\left({\left(\Delta L^{*}\right)}^{2}+{\left(\Delta a^{*}\right)}^{2}+{\left(\Delta b^{*}\right)}^{2}\right)}^{½}$$

### X-ray diffraction

X-ray diffraction was performed (D8 Advance; Bruker) with Cu Kα radiation at 40 mA and 45 kV over 15 ~ 45° 2θ, stepping 0.02° per 0.6 s. Phase identification for crystalline reaction products was by search-match software (PDXL v. 2.2.1, Integrated X-ray powder diffraction software; Rigaku, Ettlingen, Germany) against included Powder Diffraction File (PDF) data (International Centre for Diffraction Data, Newtown Square, PA, USA), verified by inspection. Patterns were obtained at each sampling time.

### Fourier-transform infrared spectroscopy

Two to five milligrams of the treated materials were ground with 100 mg potassium bromide (221864; Sigma Aldrich) using an agate mortar and pestle. Pellets were formed between dry stainless-steel plates at 1 MPa, and retrieved after a few seconds standing. Blank KBr pellets as controls were also prepared. FT-IR was performed (IRAffinity-1; Shimadzu, Kyoto, Japan) in transmittance mode over the range 400 ~ 4000 cm^-1^, with a resolution of 4.0 cm^−1^ and using 40 scans per sample.

### Electron paramagnetic resonance (EPR)

EPR spectra for 24-week materials only were obtained at ~ 9.4 GHz (X-band), with a spectrometer (EMXplus; Bruker) equipped with a resonator (4122SHQE; Bruker) and a helium flow cryostat (ESR900; Oxford Instruments, Abingdon, UK) for measurements at 12 K. (The spectra for the Dry Bi_2_O_3_ samples were measured with an ER 4118X-MD5 resonator (Bruker).) Spectra were acquired with a magnetic field sweep from 50 to 650 mT, a microwave power of 2 mW, a modulation amplitude of 0.5 mT and a modulation frequency of 100 kHz. Samples were loaded in equal volume into quartz EPR tubes (O.D. = 4 mm, I.D. = 3 mm) for the without-CO_2_ materials at both “room temperature” (RT), approximately 23°C, and 12 K. In order to assess the intrinsic response of the EPR resonators used, a background spectrum of an empty tube was also recorded under the same experimental conditions. Spectra were neither normalized nor offset to correct for sample size, focusing attention on general features rather than quantitation.

### Raman spectroscopy

Raman spectroscopy was performed on 24-week treated materials only to determine the final changes the material had undergone. Spectra were captured with a confocal Raman spectrometer (InVia; Renishaw, Wotton-under-Edge, UK), equipped with a microscope (DM 2500 M; Leica, Milton Keynes, UK) with a 20× / 0.45 NA objective lens (Nikon, Kingston upon Thames, UK), using 633 nm illumination (RL633 HeNe laser; Renishaw), an 1800 lines/mm grating and a cooled CCD camera, in software (WIRE 4.2; Renishaw).

A measure of each powder on a calcium fluoride cover glass (Crystran, Poole, UK) was scanned initially across a full spectral range of 0 ~ 3200 cm^-1^ (typical bismuth peaks below 100 cm^-1^ were not detectable due to the rejection filter design of the spectrometer). As the main Raman activity was concentrated in the region of 100 ~ 500 cm^-1^, static scans centred on 318 cm^-1^ were applied at laser power of 50% and exposure 1 s.

### Scanning electron microscopy

The control bismuth oxide, as received, and the dark powder produced after exposure to sodium hypochlorite solution for 24 weeks, were examined by SEM, both loose and in ground section. Embedding was in epoxy resin (Epofix, Struers, Ballerup, Denmark), which when set was ground using an automatic polishing machine (Phoenix Beta; Buehler, Lake Buff, USA) with progressively finer diamond discs (MD-Piano 200 ~ 1200 grit; Struers) under water, and then polished on cloth (MD-Largo, MD-Dac, MD-Nap; Struers) using diamond-impregnated polishing liquids (DiaPro 9 μm, 3 μm, 1 μm; Struers). The materials were viewed using a low-vacuum scanning electron microscope (Phenom XL; Thermo Fisher Scientific, Eindoven, The Netherlands) using backscatter mode at 10 kV.

## Results

### Purity

XRF analysis indicated no elements present except Bi and O, other than traces of Na, Si, P, Ca, Fe, and Cu. The apparent detection of (chemically implausible) 1% rubidium (Rb) is attributed to machine error, given a documented spectral interference (Table 2 in [26]). The risk of contaminant effects was therefore apparently low.

**Table 2:** Spectrophotometry. Colour change at each exposure time with respect to initial (as-supplied, ‘unexposed’) Bi_2_O_3_ control material.

Medium	Conditions		ΔE			
			1 w	4 w	12 w	24 w
**H**_**2**_**O**	light	no CO_2_	*2.2	6.2	12.5	14.9
		CO_2_	3.7	10.9	14.6	15.3
	dark	no CO_2_	2.5	5.3	6.7	5.5
		CO_2_	*1.8	11.3	5.6	5.9
**NaOH**	light	no CO_2_	10.6	14.1	21.7	18.7
		CO_2_	7.3	22.2	14.5	20.2
	dark	no CO_2_	3.5	11.9	11.0	12.8
		CO_2_	10.0	17.9	6.1	16.6
**NaCl**	light	no CO_2_	*2.2	8.2	13.6	17.5
		CO_2_	7.1	15.4	20.1	15.4
	dark	no CO_2_	3.0	*1.3	4.6	4.1
		CO_2_	7.8	13.7	5.3	8.0
**NaOCl**	light	no CO_2_	56.9	74.5	73.2	73.6
		CO_2_	63.5	74.4	72.5	77.3
	dark	no CO_2_	65.0	80.4	75.4	75.5
		CO_2_	56.5	76.5	74.1	73.3
**HCl**	light	no CO_2_	27.7	28.3	29.9	28.5
		CO_2_	27.8	28.4	28.8	27.4
	dark	no CO_2_	26.7	26.3	30.6	27.0
		CO_2_	29.1	30.9	28.6	28.4
**Bi**_**2**_**O**_**3**_	light	no CO_2_	−	−	−	28.8
	dark		−	−	−	10.4

Instances of change below “just detectable difference” marked *.

### Sample photography

**Fig 3:**
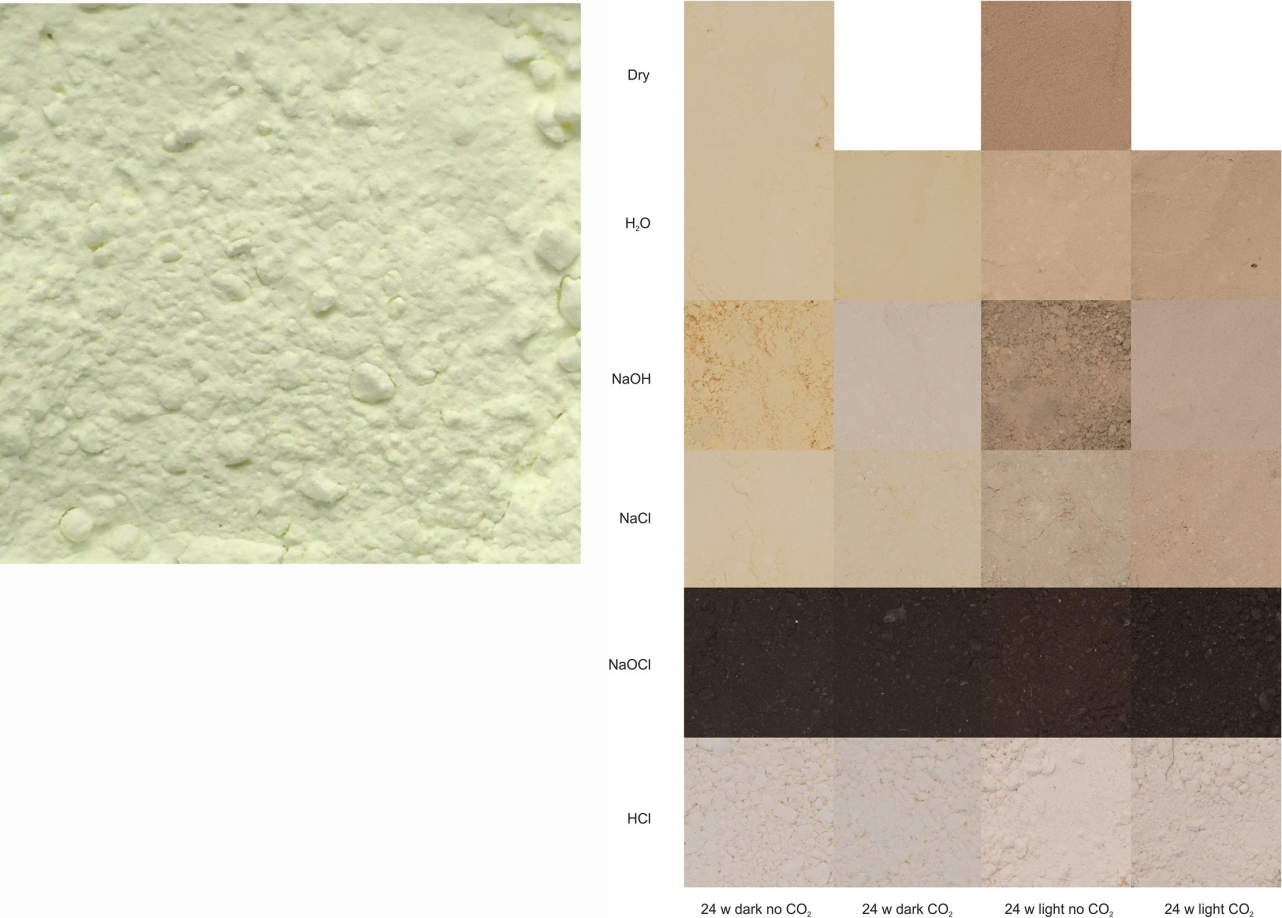
Sample photography. (a) Appearance of bismuth(III) oxide as received. (b) Appearance of treated material at 24 weeks after exposure.

**Fig 4:**
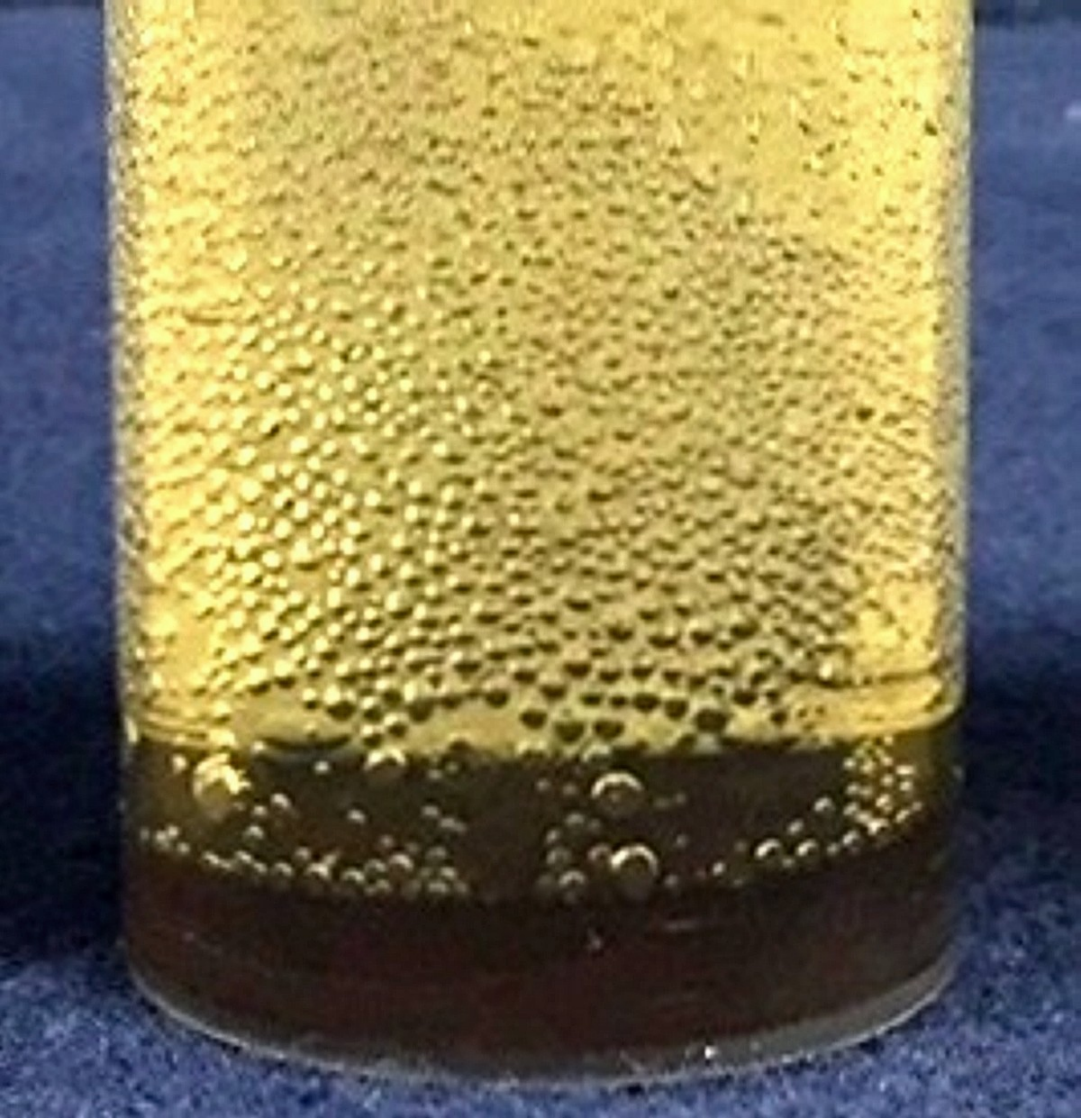
Example of gas generation. Bi_2_O_3_ in NaOCl after 24 h, Light.

The bismuth oxide as-supplied is shown in Fig 3A. The appearance of the washed and dried solids after exposure for 24 weeks, along with the dry controls, is shown in Fig 3B. The variation in discolouration between treatments is evident, as are the marked differences due to the effect of light (Fig 3B: left-hand side *vs*. right), while the effect of CO_2_ is mostly much weaker (Fig 3B: 1^st^ column *vs*. 2^nd^, 3^rd^ *vs*. 4^th^), except for NaOH. The BiOCl resulting from reaction with HCl remained pale, almost white. Reaction with NaOCl gave a very dark brown, almost black material with only some slight variation with conditions. With NaOCl reaction produced bubbles in both Light (Fig 4) and Dark, most obviously at the beginning, the gas being presumed to be oxygen on the basis of its low solubility (~0.9 mL in the 30 mL aliquant) in comparison with chlorine, which reacts at high pH to reform hypochlorite. The potential for tooth discolouration is confirmed, but reports of discolouration appear most likely to be associated with NaOCl exposure.

### Spectrophotometry

**Fig 5:**
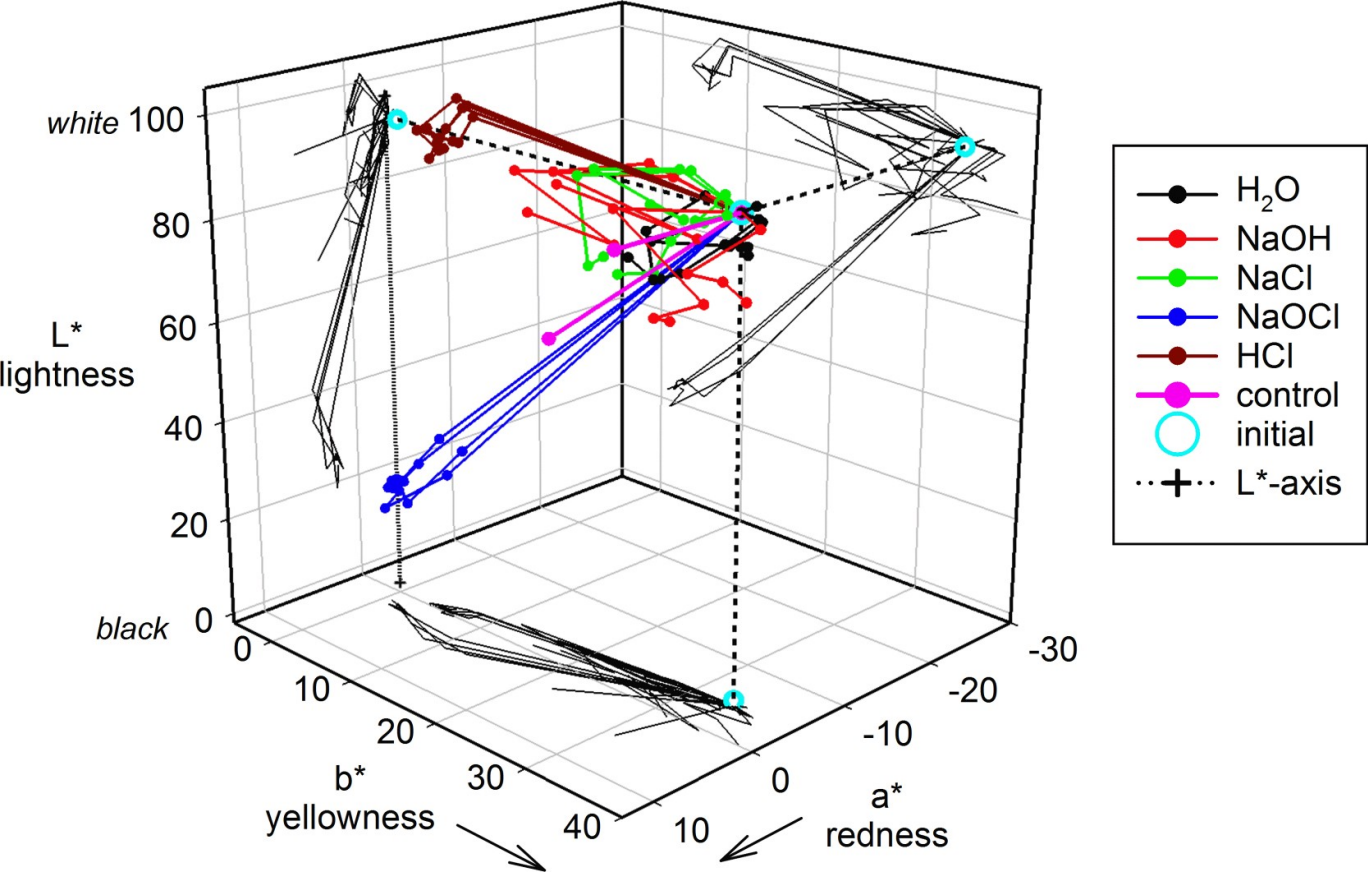
L*a*b* colour coordinate time trajectory plots. Data for all treated and exposed materials, with projections on each 2-axis plane. The control is for 24 weeks, dark and light, no added CO_2_. The L* axis (0,0,0) ~ (100,0,0) is drawn in for reference (+···+). ‘Initial’: as-supplied material.

The results of the successive spectrophotometric colour measurements on treated material over the 24 weeks are shown in Fig 5. It can be seen that the effect of HCl was to reduce b* (yellowness) to a low value with little other effect, while NaOCl caused both L* (lightness) and b* to drop promptly and markedly in value. The other media produced relatively minor but somewhat erratic changes, all of which were, however, quite visible at 24 weeks (Fig 3B), all ΔE then being greater than the “just noticeable difference” detection threshold value of ~2.3 [27] (Table 2). The distinctions for the effect of CO_2_ were mostly relatively minor, the strongest effect being seen for NaCl and NaOH in the dark, while the effect of light was most marked for H_2_O and NaCl. Discolouration therefore occurs easily, but with NaOCl the effect is dramatic.

### X-ray diffraction

There was no discernible difference between outcomes comparing the Light and Dark conditions in either the XRD or FT-IR results. That is, light had no detectable effect on the chemistry of the principal reactions. Accordingly, no further mention of this is required here, and separate data are not presented. However, those data are combined and averaged, respectively, for the XRD and FT-IR figures, to improve the signal to noise ratio.

**Fig 6:**
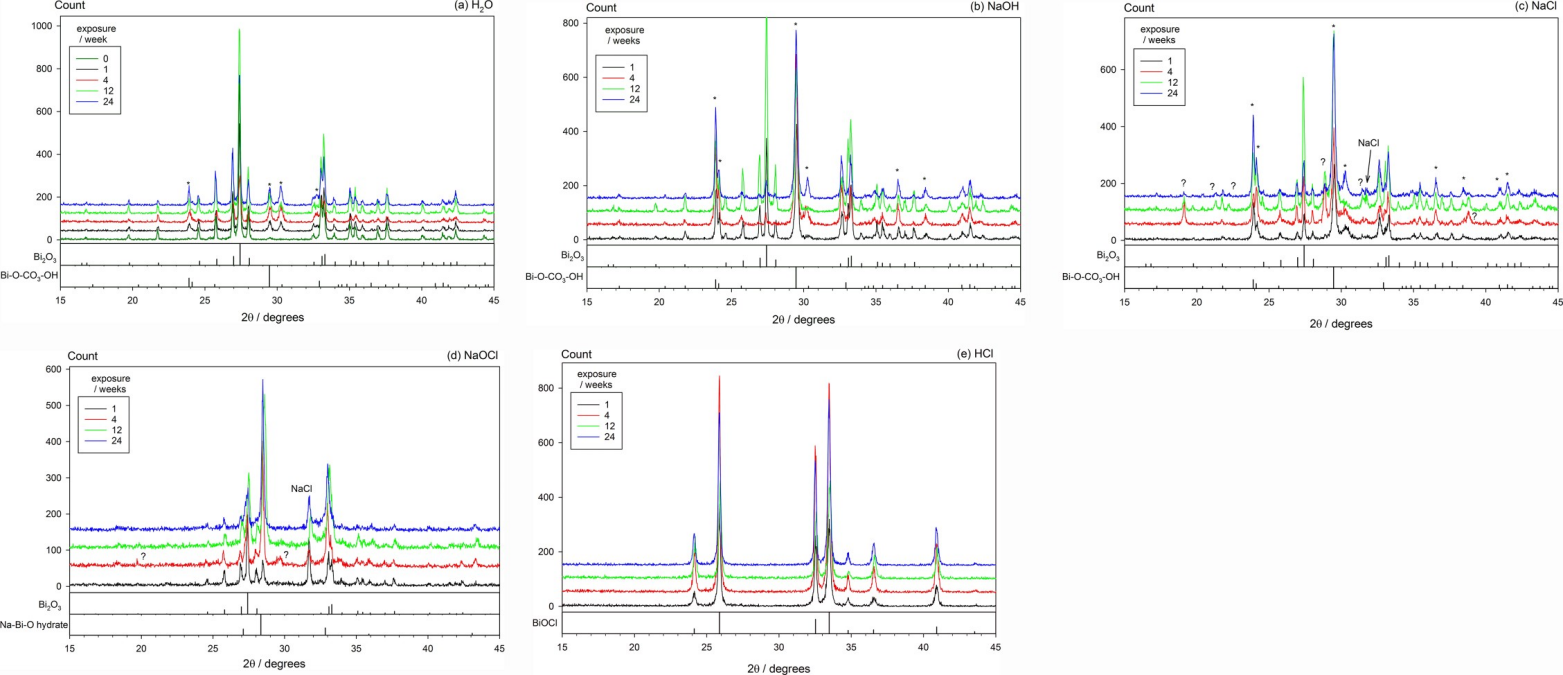
XRD patterns from treated powders exposed to added CO_2_. Patterns for Dark and Light conditions were averaged for noise reduction, there being no detectable systematic difference between the two. (a) H_2_O: the subcarbonate is present (‘*’). “0 weeks” = unexposed control. (b) NaOH: subcarbonate formed more strongly. (c) NaCl: subcarbonate again present, along with an unidentified but transient substance (‘?’); a remnant of NaCl is present. (d) NaOCl: bismuthate formed; some oxide remains; unidentified peaks marked ‘?’; a remnant of NaCl is present. (e) HCl: only BiOCl present.

The XRD results are shown in Fig 6. The untreated bismuth oxide gave the expected peaks for the monoclinic (α) form (ICDD: 00-014-0699) (Fig 6A, 0 weeks). The main peak is located at 27.37° 2θ, with other peaks of lower intensity at 25.75, 26.92, 27.99, 33.04, 33.24°.

Exposure to water + CO_2_ caused the appearance of several peaks which may be attributable to “bismuth oxide carbonate hydroxide”, (BiO)_4_CO_3_(OH)_2_ (ICDD: 00-038-0579), and which may be referred to as a ‘subcarbonate’, with the main peaks at 23.90 and 29.46° (Fig 6A). In the “CO_2_-absent” runs (not shown), contamination from the air gave only rather weak such peaks, there being no other long-term effect detectable.

Treatment with NaOH and NaCl and exposure to elevated CO_2_ also produced the subcarbonate. These peaks were apparent at week 1 and intensified with exposure, more strongly for NaCl (Fig 6B and 6C). At 24 weeks, the bismuth oxide signal was much reduced in favour of that for the subcarbonate. For NaCl “CO_2_-absent”, the same material was formed but with much lower peak intensity, while for NaOH “CO_2_-absent” there was no trace of this subcarbonate whatsoever, only the oxide being detected. This is presumably a result of the equilibrium at high pH not favouring that formation with a low CO_2_ concentration, even though contamination is more likely with the high-pH medium. However, for NaCl, unidentified peaks were found at ~19.1, 21.3, 22.1, 28.8, 31.5 and 38.8° 2θ, although by 24 weeks they had all but disappeared (Fig 6C). Slight contamination by residual NaCl was evident here, with the peak at 31.69° 2θ (ICDD: 01-080-3939).

Exposure to NaOCl resulted in the rapid appearance of strong peaks at 28.52 and 33.05° 2θ (Fig 6D), corresponding to the record for sodium bismuthate(V) hydrate, NaBiO_3_.xH_2_O (ICDD: 04-013-3875). A peak corresponding to NaCl was removed on further washing with DI. The subsequent presence of CO_2_ produced only a very weak indication of the main subcarbonate peak at ~29.46° 2θ early on, although this weakened with time of exposure to be undetectable at 24 weeks (Fig 6D).

Treatment with HCl solution only resulted in complete and rapid formation of bismuth oxychloride, BiOCl (ICDD: 01-085-0861), at 1 week, with no evidence that the subsequent exposure to CO_2_ had any effect on this, even at 24 weeks (Fig 6E).

In each case, the formation of crystalline products indicates solution-mediated reaction, and thus appreciable effective solubility of the oxide, contrary to the general assumption.

### Fourier-transform infrared spectroscopy

**Fig 7:**
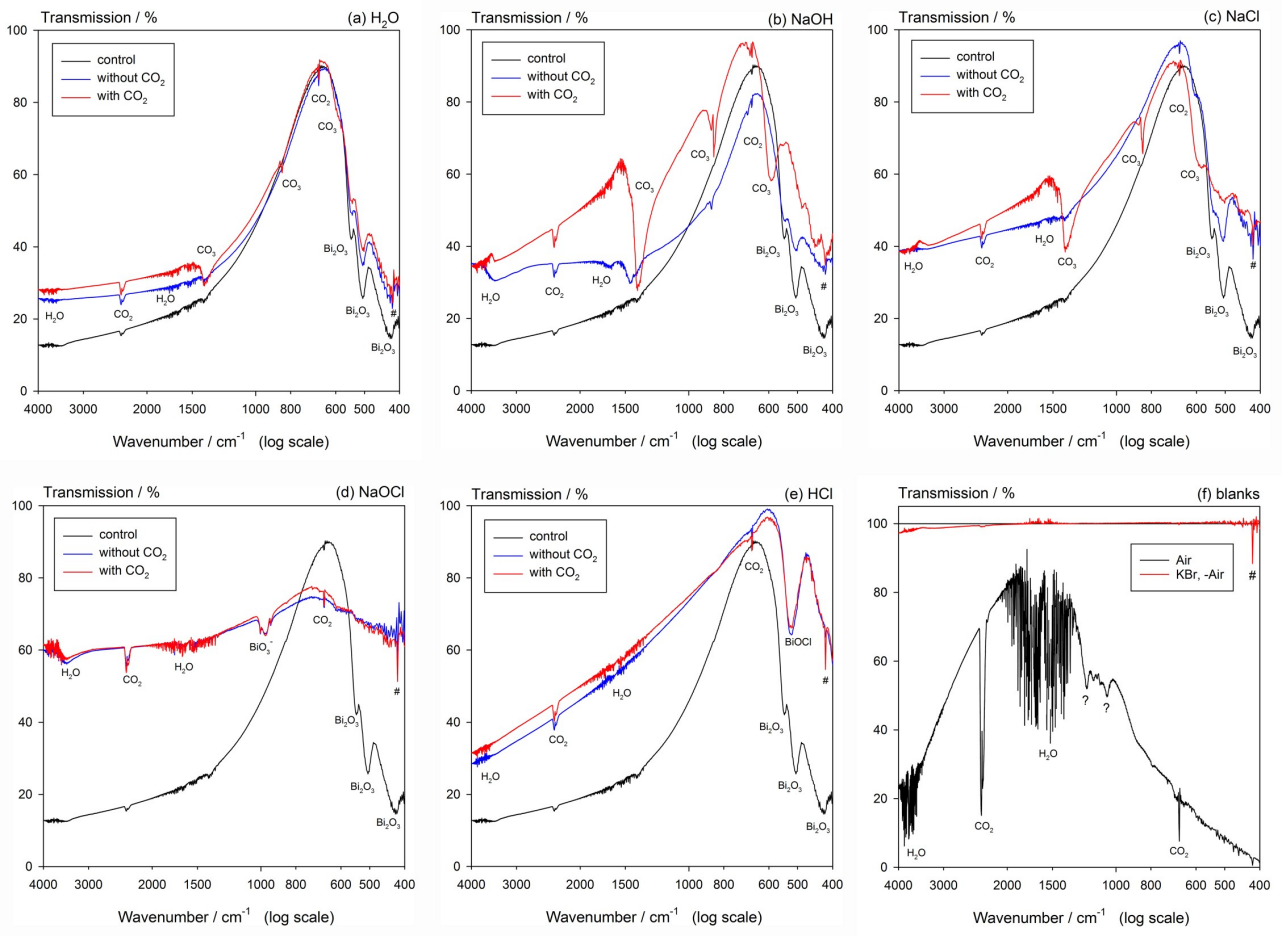
FT-IR spectra for treated materials at 24 weeks after exposure. Spectra for Dark and Light conditions were averaged for noise reduction, there being no detectable systematic difference between the two. Spectrum for Bi_2_O_3_ included for reference. Artefact marked ‘?’. Contribution from atmospheric CO_2_ incompletely cancelled. (a) H_2_O: oxide, with carbonate present. (b) NaOH: oxide still detected, but strong carbonate peaks found for added CO_2_. (c) NaCl: oxide remains, strong carbonate peaks for added CO_2_. (d) NaOCl: no oxide detectable, nor carbonate. Moderate broad peak with two satellites attributed to bismuthate. (e) HCl: only BiOCl detected. (f) Blanks: showing that KBr disc spectrum is clear after background subtraction except for the artefact at 419 cm^-1^ and unknown peaks at 1060 and 1205 cm^-1^.

The results of the FT-IR spectroscopy are shown in Fig 7A–7F. The background spectrum for KBr alone shows a very sharp feature at 418 cm^-1^. Since KBr is known to be completely transparent in the range covered, this represents either an unidentified contaminant (unlikely) or an artefact, and so can be discounted in all spectra (marked #).

Inspection of each spectrum showed no substantive changes with time in any of the features for any treatment, so the data were averaged to improve the signal-to-noise ratio. Bismuth oxide itself showed three wide bands at around 425, 505 and 543 cm^-1^. For treatment with water there was no appreciable change in these. With CO_2_, clear peaks due to this and carbonate (590, 850, 1384 cm^-1^) were then present (Fig 7A). For NaCl, the oxide bands weakened and those for carbonate strengthened (Fig 7C), with a similar but stronger outcome for NaOH (Fig 7B) as the oxide was reacted further. (The presence in all spectra of a signal from CO_2_ may be attributed to deficiencies in the background correction, as seen in Fig 7F, but may also represent adsorption, without reaction, from the air. It may be ignored.)

For HCl, apart from CO_2_ contamination, only a single wide band attributed to BiOCl was present at ~522 cm^-1^ [28], unaffected by the deliberate presence of CO_2_ (Fig 7E). Similar apparent complete reaction of the oxide was found for NaOCl, with the appearance of a moderate and wide band centered at ~971 cm^-1^, flanked by two weak but sharp peaks at ~938 and 1003 cm^-1^ (Fig 7D); these three features are unidentified (no relevant spectrum has been located), but are presumably due to the sodium bismuthate identified by XRD (above).

### Electron paramagnetic resonance

**Fig 8:**
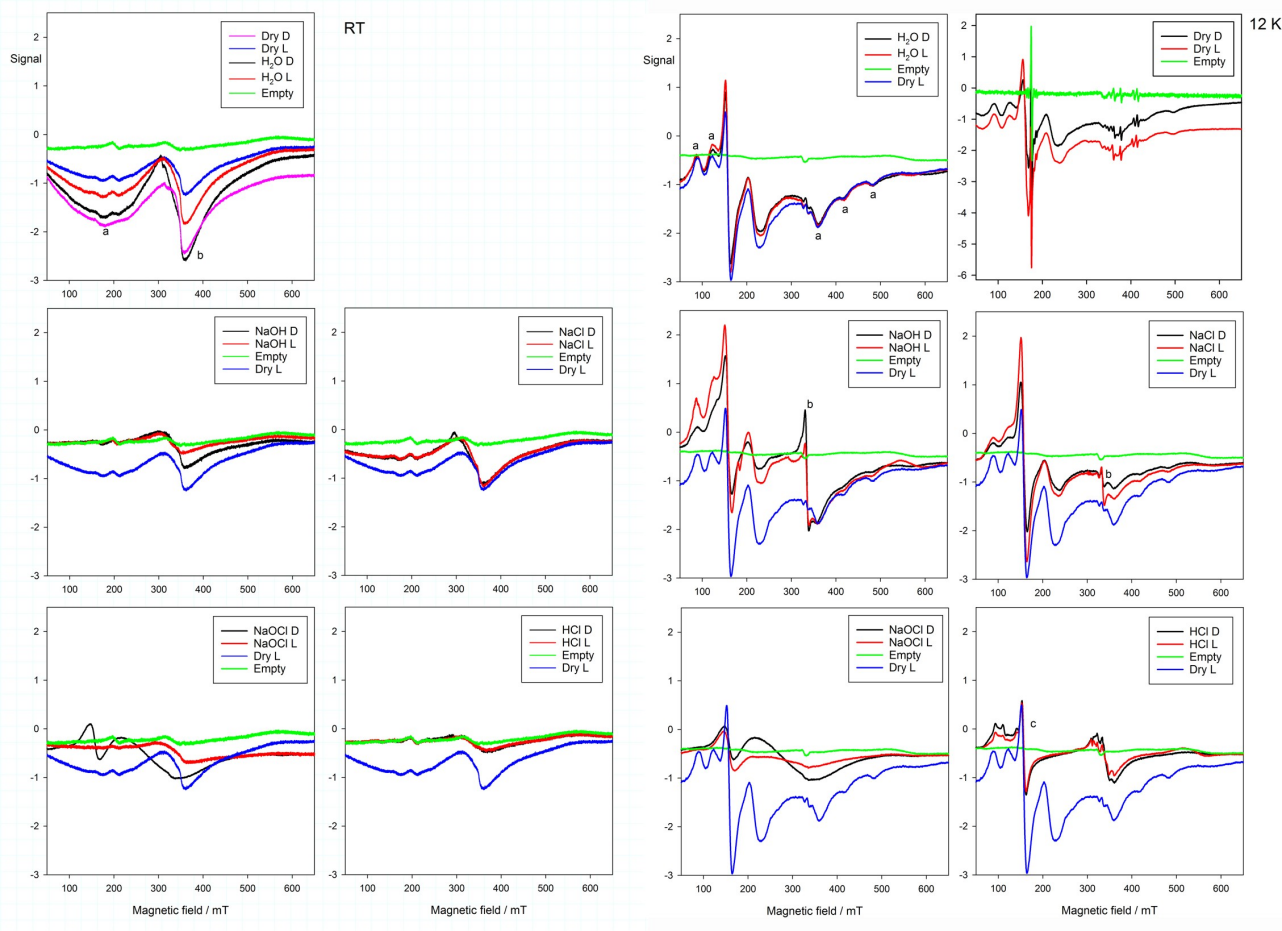
EPR spectra for treated materials at 24 weeks, exposed without added CO_2_. (a) at room temperature (RT). The principal signal is broad, and with some variation, and due to Bi_2_O_3_, except for NaOCl which shows a different kind of response, but Light resulted in almost no signal in comparison with Dark. (b) at 12 K. The principal signal is all, with small variations, due to Bi_2_O_3_, except for NaOCl where the result is obscured by metallic-like conductivity, and more so for Light than Dark.

The room temperature (RT) EPR spectra of all samples showed two broad and prominent features (labelled a and b, Fig 8A) except for the almost complete loss of these for HCl and a completely different pattern for NaOCl. At 12 K there were several strong and much sharper features (Fig 8B), including those for a putative hyperfine interaction of the paramagnetic centres with ^209^Bi (I = 9/2) (labeled ‘a’). Exposure to NaCl caused a very sharp feature (‘b’) to appear at ~335 mT (*g*~2), stronger again for NaOH, for which only a trace is seen for H_2_O. This feature was replaced by a more complex structure for HCl. A strong feature at ~158 mT (‘c’) persisted through all except NaOCl. The spectrum for NaOCl consisted of only two features of a very broad kind, the one at lower-field may correspond to ‘c’. In recording the spectra it was noted that the NaOCl sample significantly lowered the quality factor of the EPR resonator suggesting the presence of metallic-like conductivity. In light, however, the higher-field feature here was substantially weakened, but for all other treatments there was essentially no distinction between the effects of light and dark.

Since EPR is sensitive only to unpaired electrons, the signals found are suggestive of defect structures. Such defects may also be associated with the colour of substances. Low-level impurities in the oxide are another possibility.

### Raman spectroscopy

**Fig 9:**
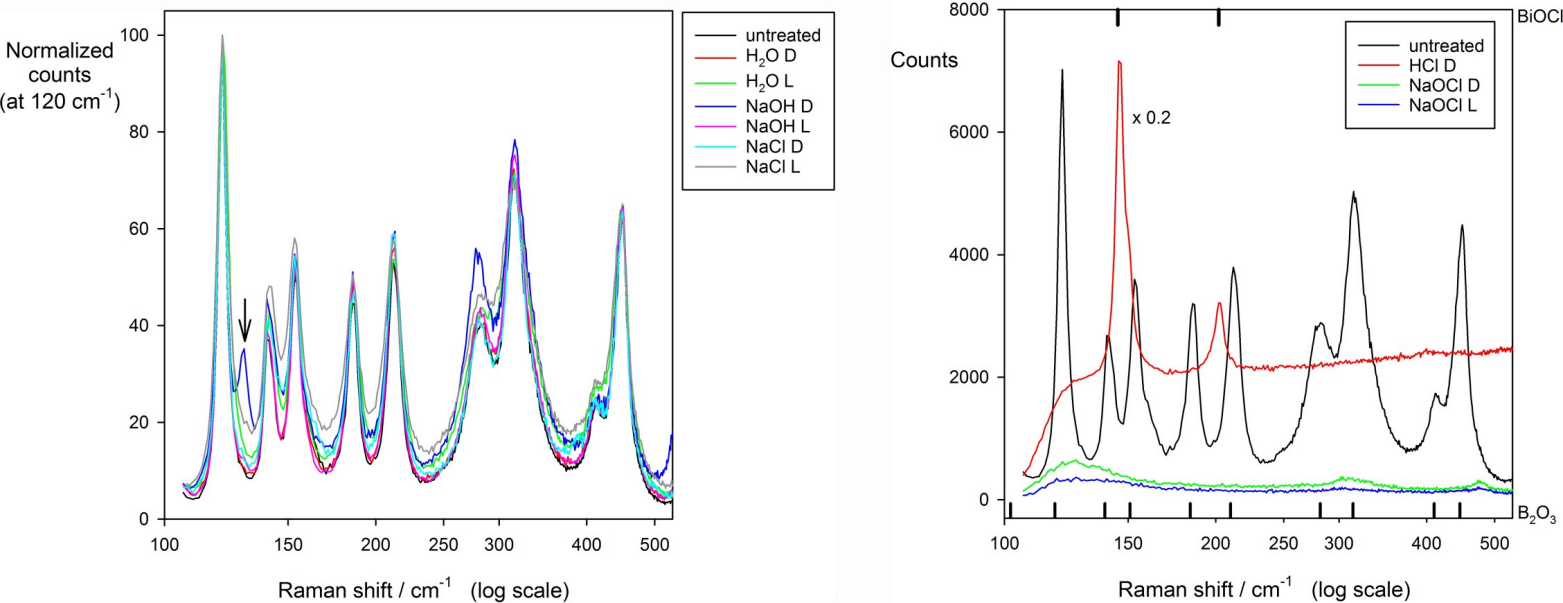
Raman spectra for treated materials at 24 weeks. Without added CO_2_, with untreated Bi_2_O_3_ for comparison. (a) For H_2_O, NaOH and NaCl. The only appreciable effect is for NaOH Dark at 130 cm^-1^. (b) For HCl and NaOCl. For NaOCl, the signal is weak and diffuse. Reference peak positions shown for BiOCl (above) and Bi_2_O_3_ (below).

Aside from variations in amplitude (presumably accountable by the reflectance of differing powder hues) the spectrum of the untreated material was matched by those of the oxide for light and dark with H_2_O and NaCl, and that treated with NaOH in light (Fig 9A). NaOH dark showed a peak at 130 cm^-1^ not apparent in other material. Subsequent area scans comprising 40 spectra of NaOH-treated material showed that this line is always present as a defined peak or a shoulder for the dark, whereas it is either absent or appears as a shoulder for the light, and also exists as a shoulder for NaCl. HCl-treated material showed huge fluorescent activity in the mid-region of full 0 ~ 3200 cm^-1^ scans, and in static scans just two well defined peaks (Fig 9B) with other peaks either not present or hidden by fluorescence.

Both light and dark NaOCl treated material show little Raman activity, with three low, broad peaks; two of these corresponding to the untreated material’s lower and middle peaks whilst the third is around 30 cm^-1^ to the right of the upper peak at 449 cm^-1^ (Fig 9B).

### Scanning electron microscopy

**Fig 10:**
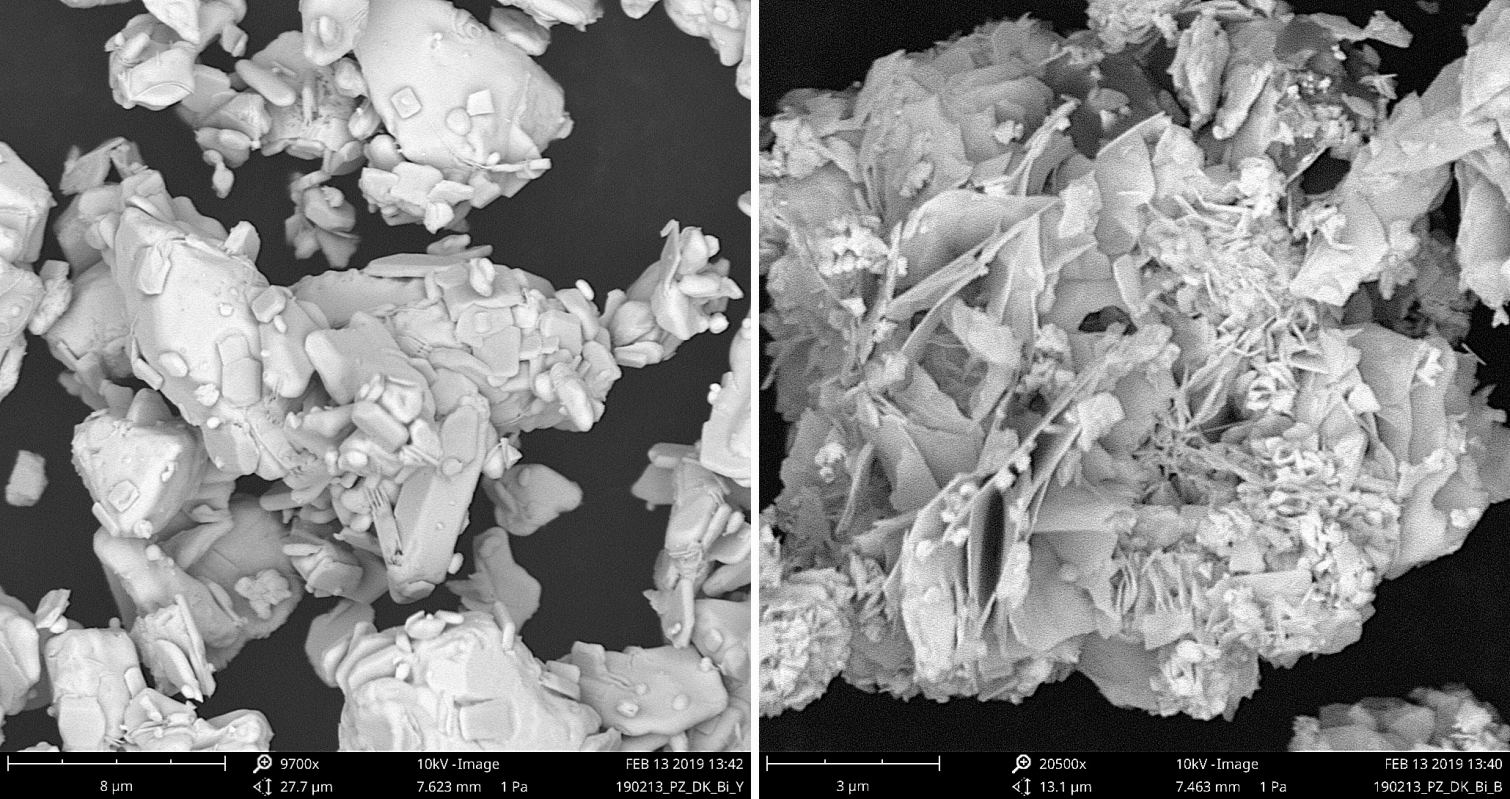
SEM micrographs. (a) bismuth oxide as-supplied, (b) reaction product for NaOCl at 24 weeks, Dark, no added CO_2_.

Micrographs of the original oxide and the product of treatment with NaOCl for 24 weeks show that crystallization from solution has occurred in forming the latter material as thinly platy rosettes and not merely a surficial transformation (Fig 10). The reaction is thus solution-mediated.

## Discussion

Bismuth compounds have long been used in root filling materials: ‘bismuth phosphate’ since at least 1952 [29], ‘bismuth subcarbonate’ (1958) [30], ‘bismuth oxide’ (1961) [31], and ‘bismuth subnitrate’ (1964) [32]–all are described as insoluble and used presumably as radio-opacifiers, although explicit documentation has not been found. The oxide at least has a number of incidental reports of apparently associated tooth discoloration [3–8]. Rapid (24 h) and marked discoloration in contact with sodium hypochlorite has been reported [10] although the reaction involved was not clear. The present choice of solutions based on sodium and chlorine was to attempt to elucidate the cause and chemistry of the discoloration of the oxide in the latter context. The 2-factor design (±light, ±CO_2_) was following reports of the colour change being associated with light in the physiological context [16, 17], although lack of oxygen was not tested.

BiOCl has long been used as a cosmetic ingredient [33] and in automotive paint [34, 35] but is known to be subject to photoreduction: Bi^3+^ ↦ Bi^0^ [36, 37], resulting in darkening. Such a reaction might reasonably be suspected in the present context. However, while exposure to HCl formed BiOCl, rapidly, as would be expected [38], essentially from the direct hydrolysis of BiCl_3_, this only showed very minor colour changes subsequently. In addition, there was no expectation or evidence that BiOCl could form other than at low pH. For both reasons it may be discounted as irrelevant to the present enquiry.

H_2_O alone had no appreciable effect on the detectable chemistry, despite the marked colour changes. NaCl produced an as-yet unidentified phase (XRD), with the main peaks at ~19.1°, 28.8° and 38.8°; this appeared to be transient, being essentially undetectable at 24 weeks (Fig 6C). This transient phase may account for the complicated colour trajectory over that time (Fig 5), but it also indicates that there are aspects of the chemistry under benign conditions that are otherwise unreported. NaOH-treated material also shows no reaction in the absence of CO_2_. However, in the presence of CO_2_, each of H_2_O-, NaCl- and NaOH-treated material all produce some bismuth subcarbonate, but this seems to have no obvious general effect on discolouration in the dark; it has been reported to be light-sensitive [39]. The mere fact that substantial reaction to form this compound after treatment with as benign a medium as normal saline with CO_2_ then present is a disturbing indicator of the reactivity of bismuth oxide, belying the assumed inertness which is the justification for its use in dental products, under an approximation to physiological conditions as well as in the context of its relevance as an endodontic irrigant.

Light increased the darkening that occurs with time for all media, including Dry, and irrespective of the presence of subsequent CO_2_. However, no chemical change was detected to account for this. Even so, the overall effect was relatively small in comparison with the dramatic effect of NaOCl–which darkening was essentially complete by 4 weeks, although it was substantial at 24 h [10]. This appears to be associated with the prompt (< 1 week) formation of a coating of sodium bismuthate, although the reaction was not complete even at 24 weeks: the Bi_2_O_3_ peak at ~27.3° is still much in evidence by XRD (Fig 6D). This substance has a very distinct FT-IR signature (note: X-radiation is penetrating, while IR is not, hence the absence of a bismuth oxide signal in Fig 7D). Given that this bismuthate has the Bi in oxidation state +5 (as opposed to the +3 of the oxide), we therefore deduce that it is the action of the oxidant hypochlorite under high pH conditions (and neither just chloride at high pH, nor the high pH itself) that generates the compound that, while nominally stable (from the persistence of the XRD pattern), spontaneously undergoes some chemical change that causes the dramatic darkening, irrespective of light exposure or the presence of carbonate. This darkening is, as far as we can tell, a novel and unexpected reaction for such a system. In addition, there is evidence of a further but transient but unidentified phase in the peaks at ~19.5°, 25.6°and 29.5°. These were not apparent at 24 weeks. This does not appear to be the same phase as occurred for NaCl.

It should be noted that as the history of the bismuth oxide was unknowable, it could not be assumed that no prior light exposure had occurred, and a true ‘dark’ control was not possible therefore, only the as-supplied material when first opened.

Previously, bismuth oxide has been reported to lack stability in alkaline environments such as that in HSCs, where leaching has also been noted [40]. Colour changes, from yellow to dark brown and black, have been found in the presence of sodium hypochlorite solution [10] and both in the presence of light and under anaerobic conditions [13, 14]. It has been postulated that bismuth oxide reacts to form a bismuth carbonate when in contact with sodium hypochlorite [10]; this now is seen not to be the case, at least, not as an end point as it remained undetected by XRD (Fig 6D). The carbonation of the bismuth oxide in air due to the presence of carbon dioxide has been postulated [10], and was said to be exacerbated by the anaerobic conditions that are present in infected teeth [13, 14]. It can now be said that for pH ~7–13, in the absence of a strong oxidant (but in the presence of atmospheric oxygen), and even without chloride, such a reaction does occur quite readily; this is enough to undermine the assertion of the inertness of bismuth oxide under mild conditions. Evidently, anaerobic conditions are not required for this.

It has in fact been reported that the reactivity of bismuth(III) oxide is such as to produce not only (BiO)_4_CO_3_(OH)_2_ but also (BiO)_2_CO_3_ when exposed to air, whether or not saturated with respect to water vapour and whether or not with added CO_2_ [41, 42], albeit slowly, such that the remark could be made that “The aging experiments demonstrate the fact that Bi_2_O_3_ is reactive under atmospheric conditions and must be tightly sealed and desiccated to avoid compositional changes [42].” The general assumption of inertness has therefore long been known to be incorrect, but awareness of this seems not to be generally understood–no reliable references to this behaviour had been encountered in preparing this study. Had it been realized at the outset, the controls used would have been more extensive. Nevertheless, the likelihood of reaction in the clinical context is underlined.

The EPR results are inconclusive. Clearly, for NaOCl the Bi environment is appreciably changed in a fashion that is not seen for any of the other treatments, which could correspond to the (+V) oxidation state. However, the signal interference from what appears to be metallic-like conductivity is suggestive of a more complicated situation than a simple reaction to a new compound, presumed to be a bismuthate. The fact that the signal is even weaker for the light-exposed material only adds to the puzzle, and the difficulty (greater metallicity?). Curiously, the Raman results are also difficult to interpret, with an almost total lack of signal for the NaOCl-treated material (again, even weaker for the light-exposed material), while the spectrum is more or less consistent with that of the oxide for all other (CO_2_-free) treatments (except for HCl, of course), although this may mostly be due to incomplete reaction.

In terms of the effect of the treatments on discolouration, it is clear that NaOCl rapidly has a very strong effect, irrespective of light exposure and the subsequent presence of CO_2_, such that if this reaction is involved clinically a marked influence on appearance might be expected. For the rest (and ignoring HCl), while the stronger effect of light on Dry Bi_2_O_3_ is noteworthy (and suggestive of a common underlying effect) it is not relevant to the conditions of use, the darkening of the oxide under both neutral and alkaline conditions (H_2_O, NaCl, NaOH) is appreciable and such as to cause concern regarding a tooth’s appearance should this be happening in a filling, which change would appear to be likely, *i*.*e*. even without the use of NaOCl.

In addition to the clinical concern with the appearance of teeth when such a material discolours or causes discolouration *in situ*, the evident reactivity under even quite mild conditions has serious implications, and is in direct contradiction of the usual view [1]. It is now seen to be feasible for some interference in the setting to occur. Bismuth ions have indeed been shown to be mobile, being found in neighbouring tooth tissue at 4 weeks [43], and in subcutaneous tissue at 3 months when implanted [13]. This diffusion is associated with tooth tissue discoloration that may be prevented by blocking the dentinal tubules with dentine bonding agent [44]. The migration of trace elements from dental cements to host tissues and essential organs, including liver, kidneys and brain, is of some concern [45–47] and requires further study.

Prevention of the darkening of bismuth oxide-containing endodontic cements and the associated tooth discolouration has been reported for the addition of 5% of zinc oxide to one brand of HSC [43]. However, the mechanism for the severe darkening then would appear to be distinct from that found here as NaOCl was not involved. Avoidance of bismuth oxide-containing materials is possible: a number of such endodontic cements and root canal sealers are available, relying on zirconium dioxide (Biodentine and BioRoot, Septodont; Totalfill sealer, FKG Dentaire), tantalum oxide (Neo MTA, NuSmile; Totalfill putty and paste, FKG Dentaire) or calcium tungstate (MTA Fillapex, Angelus) as radio-opacifier. The use of bismuth oxide in endodontic products appears therefore to be unnecessary. However, the elimination of sodium hypochlorite as an endodontic irrigant would be problematic as it is cheap and possesses high antimicrobial activity, an essential for successful endodontic therapy.

We are led to postulate that it is the remnant of treatment with hypochlorite as irrigant that can be a cause of the marked discoloration that can occur with hydraulic silicate cement containing bismuth oxide. This remnant may be present in the dentine, tubules and accessory canals and so not be directly flushable, yet emerge by diffusion subsequently. It would only require the surface of the root filling to be discoloured for the effect to be seen clinically. Even so, given the evident reactivity of bismuth oxide and the effect of light, hypochlorite is not essential for discolouration to occur, although its effect is very marked.

The identity of the reaction product with NaOCl appears to be NaBiO_3_ (of unknown hydration), but the reason for the extremely dark colour is at present obscure, given that the ordinary expectation would be that it is white or nearly so, while the commercial product of that name is off-white, yellow or brown. Further studies are in hand to characterize this material more thoroughly. Indeed, the nature of the darkening generally observed here on exposure to light does not seem to have been explained anywhere, although photoreduction must be suspected as for BiOCl.

Research continues on the use of bismuth oxide, ‘carbonate’ and subnitrate in dental materials. e.g. [48, 49], presumably on the assumption of inertness. This must now be questioned generally, given that the oxide is clearly not inert–which concern appears never to have been tested previously.

## Conclusions

Bismuth(III) oxide, as used as a radio-opacifier in a number of dental materials, shows previously unreported appreciable reactivity and discolouration under mild conditions relevant to the clinical context, despite its assumed inertness. The chemistry is evidently much more involved than hitherto suspected. The observed reactions have implications for the migration of bismuth ions into tissues. It would seem appropriate therefore to avoid its use in such contexts, indeed on the basis of these data it is considered proper to ban the use of this heavy metal in any form in all dental materials.
